# *FLI1* gene influences lesion size and skin test may predict therapeutic response in cutaneous leishmaniasis

**DOI:** 10.1590/0074-02760190361

**Published:** 2020-03-02

**Authors:** Anadilton Santos da Hora, Lucas Frederico de Almeida, Tainã Souza do Lago, Paulo Roberto Machado, Léa Cristina Castellucci

**Affiliations:** 1 Universidade Federal da Bahia, Programa de Pós-Graduação em Ciências da Saúde, Salvador, BA, Brasil; 2 Universidade Federal da Bahia, Serviço de Imunologia, Salvador, BA, Brasil; 3 Instituto Nacional de Ciência e Tecnologia em Doenças Tropicais, Salvador, BA, Brasil

**Keywords:** *FLI1*gene, therapeutic response, leishmaniasis

## Abstract

Genes associated with wound healing have been shown to be risk factors for cutaneous leishmaniasis (CL) which is caused by *Leishmania braziliensis*. In this study, we examined whether the genes previously associated with CL influenced the clinical outcome. Patients were genotyped and retrospectively classified as responders, who were cured with a single course of pentavalent antimony (Sbv), or as refractories, who did not respond to Sbv. Patients characterised as responders showed a stronger response to the leishmanin skin test (LST) when compared to the refractory subjects (p = 0.0003). Furthermore, we observed an association between the *FLI1* CC genotype and an increased size of ulcers (p = 0.0170). We suggest that the leishmanin skin test may be a predictive tool for therapeutic outcome and reinforce *FLI1* as a potential influencer of susceptibility and lesion size in CL.

American cutaneous leishmaniasis (ACL) is a parasitic infectious disease caused by a protozoa of the genus *Leishmania*. It is one of the neglected diseases having greatest impact on public health due to its globalised distribution and limitations in diagnosis, treatment, and control in endemic *foci*. Pentavalent antimony (Sbv) has been the drug of first choice for the treatment of ACL. However, there have been cumulative reports of increased resistance to Sbv in different countries.^1^ ^,^ ^2^ Individuals refractory to the treatment with Sbv have a greater initial cutaneous lesion and require longer time to heal.^3^ A previous study has demonstrated the role of wound healing genes in the resolution of CL caused by *Leishmania* spp.^4^ The friend leukemia integration 1 (*FLI1*) gene, initially identified as a gene controlling susceptibility of mice to *Leishmania major* infection,^5^ has been also associated with the development of human CL caused by *Leishmania braziliensis*.^6^ Furthermore, other lesion-healing genes linked to the *FLI1* pathway have been also associated with ACL.^7^ In this case study, we divided patients into responders and refractories based on their responses to the Sbv therapy to analyse the effects of single-nucleotide variants (SNVs), previously associated with CL, on clinical parameters. One hundred and fifty-nine cases of CL were recruited from an endemic area of Corte de Pedra, Bahia, Brazil. The diagnosis of leishmaniasis was made through a clinical examination, a positive leishmanin skin test (LST) to the soluble *Leishmania* antigen, and by detection of the parasite’s DNA through quantitative polymerase chain reaction (qPCR). Ethical approval was obtained from the IRB of the Federal University of Bahia (CEP-UFBA 22/2012) and the Brazilian National Ethical Committee (CONEP: 1258513.1.000.5537) for the use of clinical samples. Informed consent was obtained from the participants. Patients were recruited on admission at the health clinic. Blood samples were collected by venipuncture for genotyping. The patients were followed up until six months after treatment. Clinical data such as the response to treatment, the induration area for the skin test, and number and size of the ulcerated lesions were tabulated for analysis. Patients were classified as responders (controls) and refractories (cases) according to the following criteria: responders - individuals cured of CL after a single course of the Sbv standard treatment (20 mg/kg/day for 20 days) with complete ulcer cicatrisation within 90 days; refractories - patients who needed two or more courses of Sbv or an alternative drug after failure of a single Sbv treatment. Supplementary results of participants have been described in Table I. Four SNVs [*FLI1* (rs7930515), *COL1A1* (rs2586488), *CTGF* (rs6918698), and *SMAD2* (rs1792658)] were genotyped by the TaqMan^®^ qPCR technology (Thermo Fisher^®^) using predesigned genotyping assays. Analysis of the association between the SNVs and the clinical data was performed by the non-parametric one-way ANOVA Kruskal Wallis or the Mann-Whitney *U* tests using the GraphPad Prism software version 5. P values less than 0.05 were considered statistically significant. From the 159 patients, 54 (33.96%) were the CL refractory cases compared to 105 (66.04%) of the responders. We did not find significant associations between the genetic markers and the response to therapy (Table II). However, there was a strong association between the area of induration in the skin test (LST) and the treatment outcome, with the test area being significantly larger in patients responding to Sbv (p = 0.0003), as shown in Figure (A). We also observed a strong association between the CC genotype of the *FLI1* gene and the patient’s lesion size, with larger lesions observed in the carriers of this genotype (p = 0.017), as shown in Figure (B). The CC genotype was less common (C being the minor allele) and the frequencies were 11.3% and 5.1% in clinical cases and in controls, respectively. There were no significant associations between the markers and other parameters analysed.

**TABLE I:** Clinical and epidemiological features of cutaneous leishmaniasis (CL) patients according to pentavalent antimony (Sbv) response

Clinical and demographic aspects	Therapy response		
	Responders n = 105	Refractories n = 54	p value
Age, mean ± SD	27.50 ± 15.65	27,29 ± 17.18	0,7920
Gender (%)			
Male	67/105(63.80%)	32/54 (59.25%)	0,6070
Number of lesions, median, (IQR)	1 (1-2)	1 (1-2)	0,4659
Area of the greatest lesion, mm² (IQR)	120,0 (32,5 - 217,0)	132,5 (30-412,5)	0,2142
Area of Montenegro test, mm² (IQR)	300 (180 - 419)	195,5 (110 - 292,8)	0,0003
Lower limbs lesions (%)	72 (66.74)	32 (13.76)	0,3080

SD: standard deviation; IQR: interquartile range.

**TABLE II:** Logistic regression analysis between single-nucleotide polymorphisms (SNPs) and the pentavalent antimony (Sbv) response

FLI1_rs7930515	OR	CI	p
Global 2df			0.264
A/C X C/C	0.748	0.383-1.461	0.397
A/A X C/C	2.105	0.551-8.041	0.276
Global 1df			0.754
Allele A	1.080	0.662-1.763	0.755
Allele C	0.925	0.566-1.509	0.755
likelihood-radio test			0.109
COL1A1_rs2586488	OR	CI	p
Global 2df			0.946
A/G X G/G	1.079	0.565-2.058	0.817
A/A X G/G	0.922	0.319-2.662	0.881
Global 1df			0.994
Allele A	1.001	0.623-1.608	0.995
Allele G	0.998	0.621-1.602	0.995
likelihood-radio test			0.739
CTGF_rs6918698	OR	CI	p
Global 2df			0.858
C/G X G/G	0.825	0.377-1.804	0.631
C/C X G/G	0.793	0.324-1.940	0.612
Global 1df			0.619
Allele C	0.893	0.573-1.393	0.620
Allele G	1.118	0.717-1.743	0.620
likelihood-radio test			0.807
SMAD2_rs1792658	OR	CI	p
Global 2df			0.554
A/C X C/C	0.553	0.156-1.961	0.360
A/A X C/C	0.729	0.212-2.502	0.616
Global 1df			0.900
Allele A	1.031	0.637-1.667	0.900
Allele C	0.969	0.599-1.567	0.900
likelihood-radio test			0.280

Global 2df: p value at 2 degrees of freedom; Global 1df: p value at 1 degree of freedom; OR: odds ratio; CI: 95% confidence interval.

**Figure:**
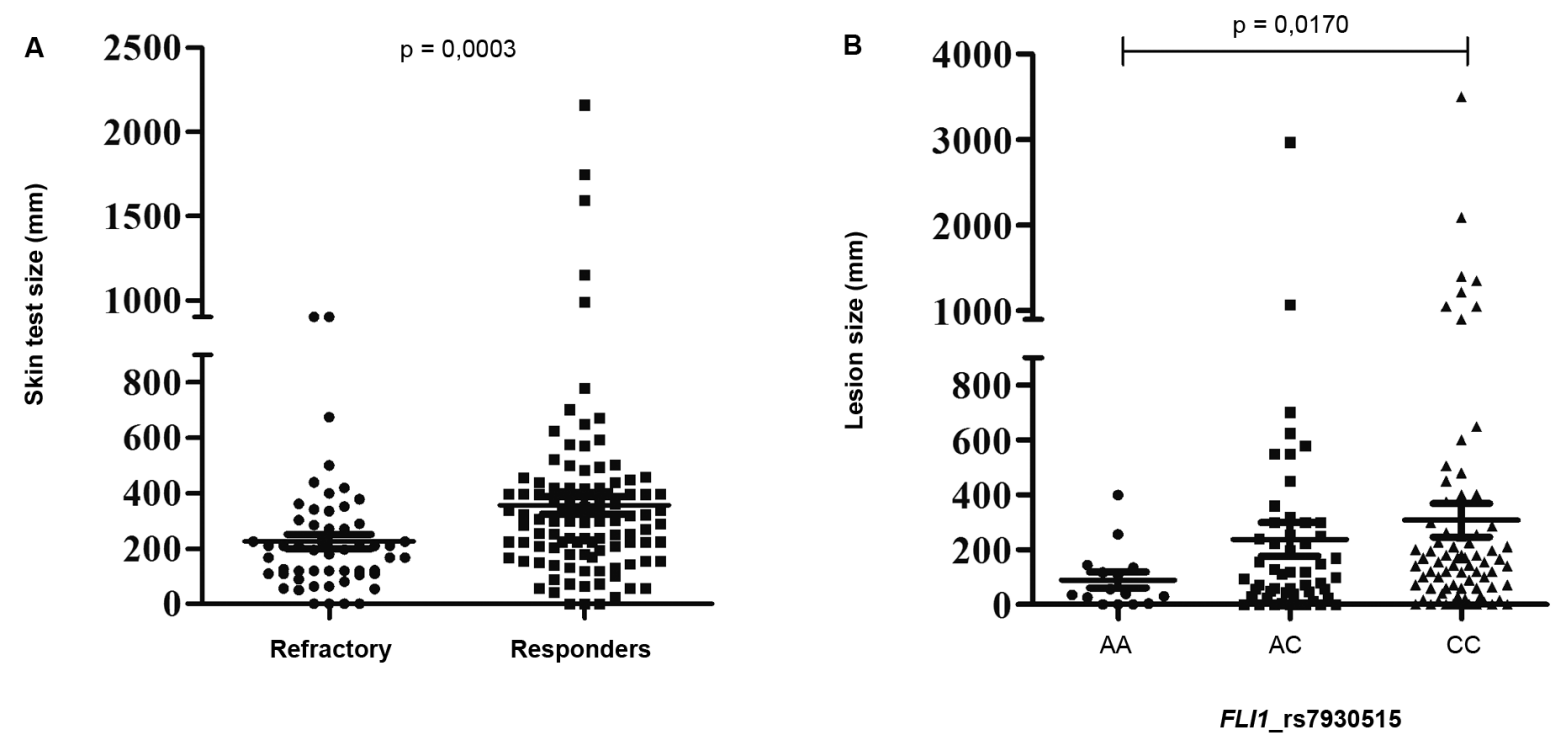
(A) Induration area (mm) in the skin test of patients classified as refractories or responders according to the treatment with a pentavalent antimony. (B) Lesion size (mm) of patients with leishmaniasis according to the genotypes of the *FLI1* gene.

The LST is one of the most common tests to diagnose CL as well as to confirm active or retrospective leishmaniasis in epidemiological surveys.^8^ Consistent with our results, a study in Brazil assessed the association between LST and therapeutic response and showed that the patients who did not respond to the treatment had less intense LST reactions than those who achieved clinical cure. For every 10 mm increase in size of the induration area in response to the skin test, there was 26% reduction in the probability of a treatment failure.^9^ The greater induration area in responding patients may be attributed to their ability to develop an effective response in the early stages of infection by producing proinflammatory cytokines and reducing cytotoxic activity. Exacerbation of the cytotoxic activity has been reported to contribute to the increase in the number and duration of the lesions.^10^ Previous results have shown that the LST stimulates IFN-γ production^11^ and this may be reflected in the ability of this group to get cured of the disease with a single course of treatment. Regarding the association between the CC genotype (rs7930515) at the *FLI1* gene and the size of the lesions, the C allele has been previously reported to be associated with an increased susceptibility to the human CL,^6^ corroborating murine studies on *L. major* resistance.^5^ In this context, our data supported the role of *FLI1* as an important gene involved in tissue repair mechanisms during human CL since the regulation of polymorphism of this gene is associated with a lesion formation. More functional studies need to be performed to evaluate the mechanisms of gene expression in patients infected with *L. braziliensis* to determine their potential as influencers of the response to treatment and to explore their role as therapeutic targets.
